# A Compend of Organic and Medical Chemistry

**Published:** 1884-12

**Authors:** 


**A Compend of Organic and Medical Chemistry**: Including Urinary Analysis and the Examination of Water and Food. By HENRY LEPPMANN, M. D., Professor of Chemistry and Metallugy in the Pennsylvania College of Dental Surgery, etc. Philadelphia: P. Blakiston, Son & Co. 1884.

The aim of this little book is to present a sufficient outline of organic and medical chemistry to serve the purposes of the medical student. It is as valuable as such compends usually are.

